# Reduced PICD in Monocytes Mounts Altered Neonate Immune Response to *Candida albicans*

**DOI:** 10.1371/journal.pone.0166648

**Published:** 2016-11-21

**Authors:** Stephan Dreschers, Peter Saupp, Mathias Hornef, Andrea Prehn, Christopher Platen, Joachim Morschhäuser, Thorsten W. Orlikowsky

**Affiliations:** 1 Department of Neonatology, University Children’s Hospital, Aachen, Germany; 2 Institute of Medical Microbiology, University Children’s Hospital, Aachen, Germany; 3 Department of Environmental Medicine, University Children’s Hospital, Aachen, Germany; 4 Department of Microbiology, University of Wuerzburg, Wuerzburg, Germany; Universitatsklinikum Freiburg, GERMANY

## Abstract

**Background:**

Invasive fungal infections with *Candida albicans* (*C*. *albicans*) occur frequently in extremely low birthweight (ELBW) infants and are associated with poor outcome. Phagocytosis of *C*.*albicans* initializes apoptosis in monocytes (phagocytosis induced cell death, PICD). PICD is reduced in neonatal cord blood monocytes (CBMO).

**Hypothesis:**

Phagocytosis of *C*. *albicans* causes PICD which differs between neonatal monocytes (CBMO) and adult peripheral blood monocytes (PBMO) due to lower stimulation of TLR-mediated immune responses.

**Methods:**

The ability to phagocytose *C*. *albicans*, expression of TLRs, the induction of apoptosis (assessment of sub-G1 and nick-strand breaks) were analyzed by FACS. TLR signalling was induced by agonists such as lipopolysaccharide (LPS), Pam3Cys, FSL-1 and Zymosan and blocked (neutralizing TLR2 antibodies and MYD88 inhibitor).

**Results:**

Phagocytic indices of PBMO and CBMO were similar. Following stimulation with agonists and *C*. *albicans* induced up-regulation of TLR2 and consecutive phosphorylation of MAP kinase P38 and expression of TNF-α, which were stronger on PBMO compared to CBMO (p < 0.005). Downstream, TLR2 signalling initiated caspase-3-dependent PICD which was found reduced in CBMO (p < 0.05 vs PBMO).

**Conclusion:**

Our data suggest direct involvement of TLR2-signalling in *C*. *albicans*-induced PICD in monocytes and an alteration of this pathway in CBMO.

## Introduction

Although *C*. *albicans* is part of the normal microbiota, it may emerge as opportunistic pathogen in the event of innate or acquired immunodeficiency. *C*. *albicans* and other *Candida* species are also common pathogens in preterm infants. Very low birthweight (VLBW) and extremely low birthweight (ELBW) infants are extremely vulnerable to invasive *Candida* infection (ICI) with adverse neurodevelopmental outcome and high mortality. The spectrum of clinical disease comprises mucocutaneous infections, blood stream infection and invasive infections of various internal organs [1, 2].

Preterm infants exhibit a particular sensitivity to fungal infections. These opportunistic pathogens take advantage of the immature neonatal immune system and the need for prolonged antibiotic treatment that facilitates fungal growth, replication and dissemination [3]. *C*. *albicans* accounts for 9.8% of early onset sepsis (EOS) in newborns and additionally significantly contributes to neonatal late-onset sepsis (LOS), affecting approximately 7% of VLBW infants before hospital discharge and, varying from country to country up to 20% of ELBW [4]. *Candida* thereby represents the fourth most common species of LOS. There is growing evidence, that the epidemiology of *Candida*-infection still may be underestimated [3].

Insight in the pathogenesis of *Candida* infection of the neonate is required to understand the associated morbidity and mortality and develop new preventive and therapeutic strategies. *C*. *albicans* grows in different morphological forms (yeast, pseudohyphae, and hyphae) dependent on the tissue milieu. *C*. *albicans* ability to grow as hyphae was shown to contribute enhanced pathogenicity and virulence [3].

At least three groups of innate immune receptors are involved in the recognition of *C*. *albicans*, namely toll-like receptors (TLRs), C-type lectin receptors (CLR) and nucleotide-binding-domain-leucine-rich-repeat-receptors (NLRs) [5]. Among these receptors, TLR2 is one of the key receptors that play a critical role in recognition of pathogens and activation of host innate immune signalling. Although the signalling mechanisms following infection are not fully understood [6,7] [8], they significantly contribute to host immune responses during candidiasis [9]. The individual host response is most likely due to differences in the innate immune recognition and the release of pro-inflammatory cytokines and chemokines [10, 11]. The downstream signalling of TLR 2 is modulated by the formation of heterodimers with TLR1 and TLR6 [12]. Stimulation of TLR2 leads to the recruitment of the adaptor molecule MYD88 [13] and the IL-1 receptor-associated kinases IRAK-1 and -4 activating a cytosolic signalling cascade that culminates in the stimulation of mitogen activated protein (MAP) kinases and nuclear factor-κB (NF-κB). Both transcription factors induce the expression of cytokines with pro- and anti-inflammatory activity. During infection, TLRs act as double edged sword triggering antimicrobial host responses but also apoptotic pathways [14].

We previously identified an involvement of the CD95/CD95L and TNF-α-pathway in the phagocytosis-induced cell death of monocytes, following infection with *E*. *coli *[15, 16]. Unexpectedly, we noted a significantly reduced PICD in neonatal monocytes despite a similar phagocytic capacity. This reduced PICD was due to defects in the CD95-/CD95L-pathway [15].

Aim of the present study was to extend our work to investigate the fungal infection with specific objectives, (i) whether the phagocytic index differs, (ii) whether TLR2 is involved in the recognition of *C*. *albicans* by monocytes, (iii) whether differences in immune recognition influence the occurrence of PICD and (iv), whether PICD of neonate or adult monocytes is inhibited by manipulation of the TLR2-pathway. TLR agonists were used to investigate the general responsiveness of neonatal (CBMO) versus adult (PBMO) monocytes.

## Material and Methods

### Patients

The study protocol was approved by the Ethics Committees of Aachen University Hospital. All mothers gave written consent before they went into labour. Randomly selected, unrelated, healthy adults donated blood and served as controls. All term neonates were delivered spontaneously and did not exhibit signs of infection, as defined by clinical status, white blood cell count and C-reactive protein. Mothers with amnion infection and prolonged labour were excluded. Umbilical cord blood was placed in heparin-coated tubes (4 IE/ml blood), immediately following cord ligation.

### Reagents

Antibodies to CD14 (MEM18), CD3 (UCHT1), CD4 (RPA-T4), CD8 (SK-1), TLR2/CD282 (11G7), TLR4/CD284 (HTA125) and Ig-matched controls (IgG1, IgG2b) were from BD Biosciences and Immunotools (Heidelberg, Germany and Friesoythe, Germany). Antibodies specific for cleaved caspases were purchased by Perbio Science (Rockford, IL, USA; clone S.147.8) and New England Biolabs (Frankfurt, Germany). The secondary biotinylated anti-mouse antibody was from eBiosciences (San Diego, CA, USA). Streptavidin-Alexa Fluor 647 for detection of the biotinylated antibody, secondary antibodies to detect primary anti-cleaved- caspase antibodies and fetal calf sera (FCS) for Fc-receptor blocking were purchased from Invitrogen (Carlsbad, CA). Diamidino-2-phenylindole-dihydrochloride (DAPI) was from Merck (Darmstadt, Germany). Propidiumiodide (PI), *E*. *coli* LPS and antibiotics were purchased from Sigma (Munich, Germany). Phosphate buffered saline (1x PBS) was from Sigma-Aldrich (Munich, Germany). 4% Sabauroud-Dextrose-Agar, NaCl, Paraformaldehyde (PFA) for fixation and Trition X-100 for permeabilisation were purchased from Merck (Whitehouse Station, NJ). The TLR agonists (Zymosan, FSL-1 and Pam3Cys) were from InVivoGen (Toulouse, France). The TUNEL staining kit was purchased from Roche (Indianapolis, USA). The MYD88 inhibitory protein and the corresponding control peptide were purchased from NOVUS biologicals (Abingdon, UK). For the immunoblot analysis 6x10^6^ cells were subjected to SDS-PAGE which was performed according to standard protocols. For Imaging and quantification a LAS 3000 imager (Fujifilm, Düsseldorf, Germany) combined with the Multi-Gauge software (Fujifilm, Düsseldorf, Germany) was used. The TNF-α ELISA kit was purchased from eBbiosciences (Ebiosciences-Natutec, Frankfurt, Germany)

### Yeasts

*C*. *albicans* SC5314 (wildtype) and SCADH1G4A, a GFP-expressing strain [17, 18], kindly provided by Prof. Joachim Morschäuser (University of Würzburg, Germany), were used for stimulation- and phagocytosis-assays. Both strains were freshly grown overnight on 4%-Sabouraud-Dextrose-Agar (Merck) at 28°C and stored at 4°C during the day. Yeasts were taken from the plate, diluted in 0.9% NaCl solution and brought to 1x10^7^ cells/ml by a McFarland density index, which correctness was routinely checked by a Neubauer hemocytometer.

Two experimental challenges had to be considered performing *in-vitro C*. *albicans* infection. Infection at 37 C° leads to overgrowing yeasts. Co-cultivation of leukocytes and *C*. *albicans* at RT initiates hyphae growth which rules out any FACS analysis. Therefore, experiments were restricted to 4 hours p.i. at 37 C° followed by fixation.

Microscopical analysis revealed that both, CD14-positive PBMO and CBMO were capable to incorporate the 4–10 μm sized yeast blastospores within the selected time interval, independent of the temperature of cultivation.

### Mononuclear cell cultures

Peripheral blood and cord blood mononuclear cells (PBMO and CBMO) were isolated by density gradient centrifugation on Ficoll-Paque PLUS (GE Healthcare, Little Chalfont, UK) as described previously [19]. Washed cells were resuspended in RPMI-1640 (Invitrogen, Carlsbad, CA). For analysis of post-phagocytic reactions, cells were counted in a Neubauer hemocytometer, placed at 1x10^6^ cells/ml in flat bottom 24 well cell culture plates (Costar, Bodenheim, Germany) containing 10% heat-inactivated fetal calf serum (FCS) and 1% Penicillin/Streptomycin (both Invitrogen) and incubated at 37°C.

### Staining procedures and flow cytometry

Because of infectiousness of *C*. *albicans* the samples were fixed with 2% paraformaldehyde (1:1) for 30 min and washed with PBS + 3% FCS prior to FACS analysis. Monocytes were gated by forward (FSC), side scatter (SSC), and CD14 expression. Lymphocytes could be identified by their ability to show a characteristic CD3/CD4/CD8-positive population in FACS analysis.

TLR2- and TLR4-antibodies were added for 20 min at RT in 1:500 dilution prior to fixation. After fixation for 1 hour at RT, cells were washed twice in PBS + 3% FCS prior to FACS analysis (S1C Fig). For the detection of the cleaved forms of caspase-3 and -8, fixed leucocyte samples were permeabilized with 0.1% (v/v) Triton-X-100 in PBS for 5 min, washed twice in PBS/FCS (1% v/v) and incubated with the appropriate antibodies, diluted 1:100 for 30 min at RT. Again, samples were washed twice in PBS/FCS and incubated with the secondary antibodies, diluted 1:1000 for 5 min at RT, followed by subsequent washing. As a control, samples were incubated with secondary antibodies only or incubated with non-specific isotype control (S1D Fig). For intracellular staining, utilizing anti-TNF-α and anti-phosphorylated-P38 antibodies, cells were permeabilized by PERM/WASH buffer supplied by Becton Dickinson Biosciences (Franklin Lakes, New Jersey, USA) for 20 min at RT. The antibodies named above or corresponding isotype controls were diluted 1:200 in PERM/WASH buffer and incubated for 1 h at 4°C. After subsequent washings, cells were analyzed via FACS. The TUNEL activity staining was performed according to the manufacturer`s recommendations and as previously described [16]. To avoid false-positive signals due to interference with staining of yeast genome, we corrected our gating strategy (S1A and S1B Fig). A daily calibrated FACS-Canto II flow cytometer (Becton Dickinson, MountainView, CA) was used to perform phenotypic analysis.

### Stimulation and blocking of TLR and MYD88

In indicated experiments, the culture medium was supplemented with TLR agonists: Pam3Cys (1 μg/ml; TLR2/TLR1 agonist), LPS (10 ng/ml, TLR4 agonist), FSL-1 (1μg/ml, TLR2/TLR6 agonist), and Zymosan (5 μg/ml, TLR2/TLR6, Dectin-1). In general, stimulation intervals were identical to infection intervals and lasted 2 hours. In an *ex-ante* experiment, the agonist Pam3Cys was given for shorter and longer intervals to determine the onset of intracellular signalling (S3 Fig).The TLR2 blocking procedure followed the protocol of the manufacturer (InVivoGen, Toulouse, France). In brief, to 2x10^6^ PBMC and CBMC the antibody (PAb-hTLR2) was added 15 min prior to infection or addition of agonists in a final concentration of 5μg/ml. In control assays an equivalent concentration of non-specific antibody (Rat PAb Control) was added. Mononuclear cells were cultivated under standard conditions.

The MYD88i blocking peptide and the corresponding control peptide, consisting of the *antennapedia* region only, were administered two hours before infection and stimulation in a final concentration of 20μM. In precursory phagocytic assays we checked that incubation with either blocking antibody (TLR2 bAb) and MYD88i did not interfere with phagocytosis.

### Phagocytosis assay

Phagocytosis assays were performed by adding GFP-expressing yeasts (SCADH1G4A) in different concentrations to 1x10^6^ cells. Infection was performed at a multiplicity of infection (MOI) of 1:1 to 1:10 which was achieved by dilution with 0.9% NaCl. The phagocytosis index (CD14+GFP+ monocytes: CD14+ monocytes) and the phagocytic capacity (PC, mean GFP (MFI)) were analyzed.

### Hypodiploid nuclei

DNA fragmentation was assessed according to Nicoletti [20] and previously described [16]. In brief, mononuclear cells were stained with CD14 antibody for 15 minutes at RT to identify monocytes. A fixation with paraformaldehyde (2% v/v in PBS) for 30 min at RT was performed. Afterwards, cells were permeabilized by incubation in PBS-T (PBS, Triton X-100 0,1% w/v) for 20 minutes at RT, washed twice in PBS, resuspended in PBS-PI (PBS, 70μg/ml PI and 13 units RNAse) and incubated for 10 minutes at RT before analysis by flow cytometry.

### Cytokine detection

The IL-6 Elecsys® IL-6 (Interleukin-6) assay (Roche Diagnostics GmbH; D-68305 Mannheim, Germany) were used according to the manufacturer`s recommendations for analysis of IL-6 levels in the cell supernatant fluid. The TNF-α ELISA was used according to the manufacturer`s recommendations. The read-out was executed in a spectra max 340PC ELISA reader (molecular devices, Sunnyvale, CA, USA) with a sensitivity from 4 – 500pg/ml.

### Statistical analysis

Results are expressed as mean +/- standard deviation. Error bars represent standard deviations. Statistical analysis was performed using a student’s t-test and 1- or 2-way ANOVAs, respectively. Results of 2-way-Anova calculation were validated with a Bonferroni post test. Values of p < 0.05 were considered significant. Analyses were done with statistical software (GraphPad Software Statistical Package, La Jolla, CA 92037 USA).

## Results

### Phagocytic properties of PBMO and CBMO are similar after infection with *C*. *albicans*

**Fig 1:**
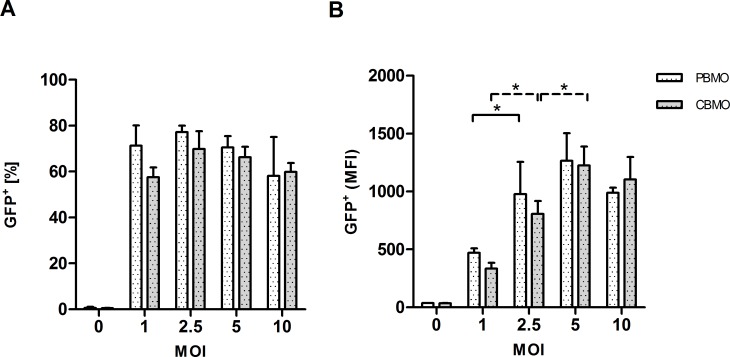
Phagocytic properties of neonatal and adult monocytes. Comparative flow cytometric analysis of the PI and PC of gated CD14^+^ monocytes in CBMO and PBMO following infection with GFP^+^ *C*. *albicans* for 2h at the indicated MOI (n = 3; student`s t-test, * p < 0.05).

We assessed the dose dependent uptake of *C*. *albicans* by FACS analysis and quantified the phagocytosis index (PI) and phagocytic capacity (PC) of monocytes, as previously described for bacterial infection assays [21] (Fig 1A and 1B). PBMO and CBMO revealed no significant differences in PI after co-incubation with increasing numbers of fungi with a MOI varying between 1 and 10 (Fig 1A). Analysis of the PC revealed that increasing numbers of *C*. *albicans* saturated the phagocytic process at a MOI of 2.5 for PBMO and at a MOI of 5 for CBMO (Fig 1B). At a MOI of 5, the PC was equal in PBMO and CBMO.

### *C*. *albicans* infection regulates surface expressions of TLR2 and TLR4

**Fig 2:**
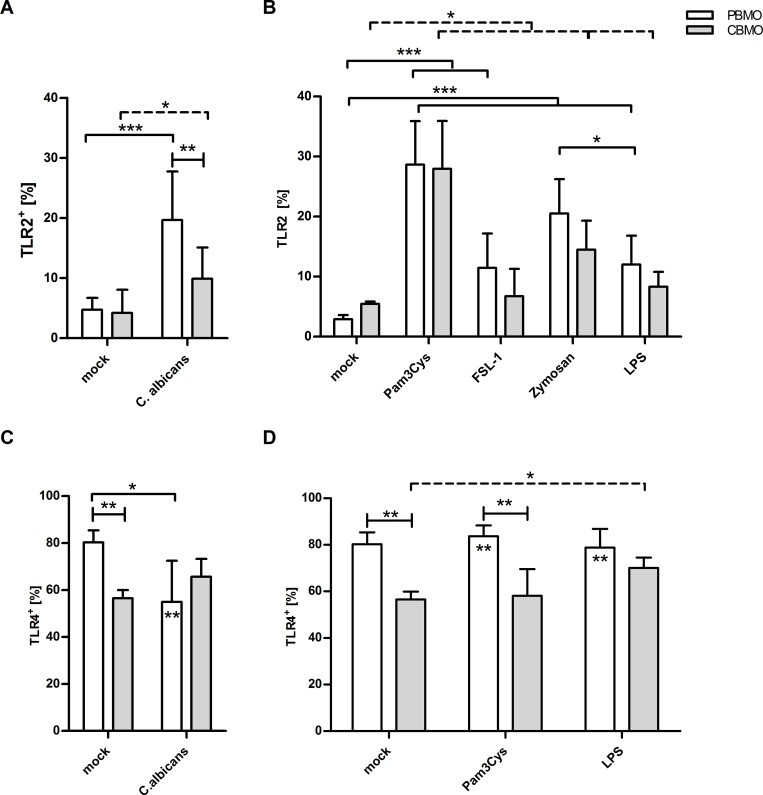
TLR2 and TLR4 surface expression by neonatal or adult monocytes following infection with *C*. *albicans* or TLR2 stimulation. CD14^+^ CBMO and PBMO were infected with *C*. *albicans* at a MOI of 5 for 2h (A, C), or treated with indicated TLR2 agonists (B, D). The surface expression of TLR2 (A, B) and TLR4 (C, D) were assessed by flow cytometric analysis (n = 8; 1-way-ANOVA test marked by crotched solid (between PBMO groups) and dotted (between CBMO groups) lines, *p<0.05; **p<0.01, ***p<0,005; 2-way ANOVA test marked by blunt ended lines and asterisks in chart bars testing groups in C and D; **p<0.01).

We assessed the basal cell surface expression of TLR2 and TLR4 and followed it after *C*. *albicans* infection and incubation in the presence of TLR2 and TLR4 agonists (Fig 2). TLR2 and TLR4 expression was modulated differently in PBMO and CBMO by *C*. *albicans* and TLR-agonists. Basal expression of TLR2 was low in CBMO and PBMO with slightly more TLR2 expression on CBMO as compared to PBMO (Fig 2A). *C*. *albicans* infection raised the number of TLR2-expressing PBMO significantly stronger as compared to CBMO (Fig 2A). Stimulation with the TLR2 agonists Pam3Cys or Zymosan resulted in almost identical TLR2 up-regulation on PBMO and CBMO.

The TLR4 ligand LPS also induced a slight up-regulation of TLR2 but to a weaker extent as compared to Pam3Cys and Zymosan (Fig 2B). FSL-1, a TLR2/TLR6 agonist, induced TLR2 up-regulation comparable to what was observed after LPS stimulation (Fig 2B). The results indicate that *C*. *albicans* and the TLR2/TLR1 agonist Pam3Cys, but not the TLR4 and the TLR2/TLR6 agonists LPS and FSL-1 efficiently up-regulate TLR2 expression.

Compared to TLR2, surface expression of TLR4 on PBMO was significantly higher as compared to CBMO (Fig 2C). Infection with *C*. *albicans* down-regulated TLR4 on PBMO, but had no significant effect on CBMO (Fig 2C). LPS up-regulated TLR4 on CBMO but had no effect on the high basal expression of TLR4 on PBMO (Fig 2D). The TLR2 agonist Pam3Cys did not change the TLR4 expression pattern (Fig 2D). These results suggest that *C*. *albicans* infection influences TLR2 expression more profoundly than TLR4 expression on PBMO. The expression of TLR2 and TLR4 (MFI) were found similar before and after stimulation in PBMO and CBMO (S2 Fig).

CD14, which serves as a co-receptor for TLR4 and TLR2 [22], was stronger down-regulated on PBMO as compared to CBMO after *C*.*albicans* infection decreasing within 2 h p.i. (196 ± 52.5 MFI to 121.3 ± 28.9 MFI; p < 0.05). CD14 expression in non-infected CBMO was lower compared to PBMO (118 ± 35.6 MFI; p < 0.05) and exhibited a weaker decline after infection (90.3 ± 8.8; p < 0.05 vs. non-infected).

### Induction of P38 MAP Kinase (MAKP) is TLR2 dependent and differs in CBMO and PBMO

**Fig 3:**
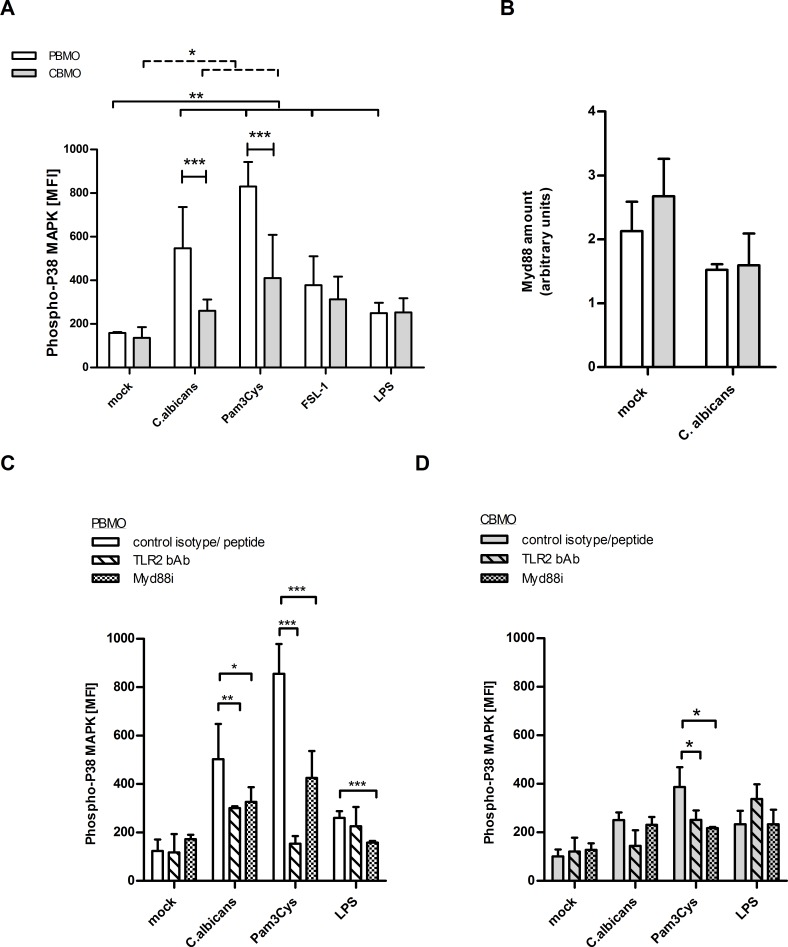
*C*. *albicans* mediated P38 MAPK stimulation in neonatal and adult monocytes. CD14^+^ CBMO or PBMO were infected with *C*. *albicans* at a MOI of 5 for 2h or treated with the indicated TLR agonists. (A) Phosphorylation of P38 MAPK was assessed by intracellular FACS staining (n = 7; 1-way-ANOVA testing marked by solid crotched (PBMO groups) and dashed crotched lines (CBMO groups), *p<0.05; **p<0.01; 2-way ANOVA test marked by blunt ended lines, ***p<0.005). (B) MYD88 protein expression was quantified by immunoblot (n = 4). (C and D) TLR2 bAb and MYD88i peptide were used to identify the role of TLR2/MYD88 signalling for P38 MAPK stimulation (n = 5; 2-way ANOVA test, *p<0.05; **p<0.01, ***p<0.005).

To monitor cellular signal transduction downstream of TLR2 and TLR4, we assessed the phosphorylation of the P38 MAPK (Fig 3). Within an infection interval of 2 h, the P38-phosphorylation in PBMO increased 2-fold stronger than in CBMO (Fig 3A, p < 0.05). Comparable results were obtained after stimulation with the TLR2/TLR1 agonist Pam3Cys (Fig 3A).

The TLR2/TLR6 agonist FSL-1 and the TLR4 agonist LPS caused a similar, but weaker P38 phosphorylation (Fig 3A), suggesting that C. *albicans* induced P38 phosphorylation primarily signals via TLR2/TLR1.

The potential to phosphorylate P38 MAPK was not due to a critical reduced in the expression of the adaptor protein MYD88. MYD88 was found to be abundantly expressed in both PBMO and CBMO before and after *C*. *albicans* infection (Fig 3B).

Blocking of TLR2 with an inhibitory Ab (Fig 3C, PBMO; Fig 3D, CBMO) reduced the *C*. *albicans-* and Pam3Cys- induced P38 phosphorylation by 50% in PBMO (Fig 3C, first and second column). In contrast, the *C*. *albicans*-induced P38-phosphorylation in CBMO was lower than in PBMO (p < 0.05) and was not abolished in the presence of the TLR2 blocking Ab (Fig 3D). This was not due to an impaired signalling via TLR2/TLR1, since Pam3Cys induced P38 phosphorylation could readily be abolished by the TLR2 bAb (Fig 3C). The TLR2 bAb did not inhibit LPS induced P38 phosphorylation in PBMO and CBMO, confirming the specificity of the blocking antibody.

Inhibition of MYD88 resulted in a decreased P38 phosphorylation after *C*. *albicans* infection or Pam3Cys stimulation of PBMO (Fig 3C). In CBMO, MYD88 blockage decreased the P38 phosphorylation after Pam3Cys stimulation, but not after *C*. *albicans* infection (Fig 3D).

In summary, these results suggest that *C*. *albicans*-induced P38 phosphorylation in PBMO occurs predominantly via TLR2/TLR1 and MYD88. Interestingly, Pam3Cys administration in CBMO was accompanied with a lower concentration of phosphorylated P38 compared to PBMO (Fig 3), although Pam3Cys up-regulates TLR2 equally in CBMO and PBMO (Fig 2).

### TNF-α expression and IL-6 secretion following infection with *C*. *albicans*

**Fig 4:**
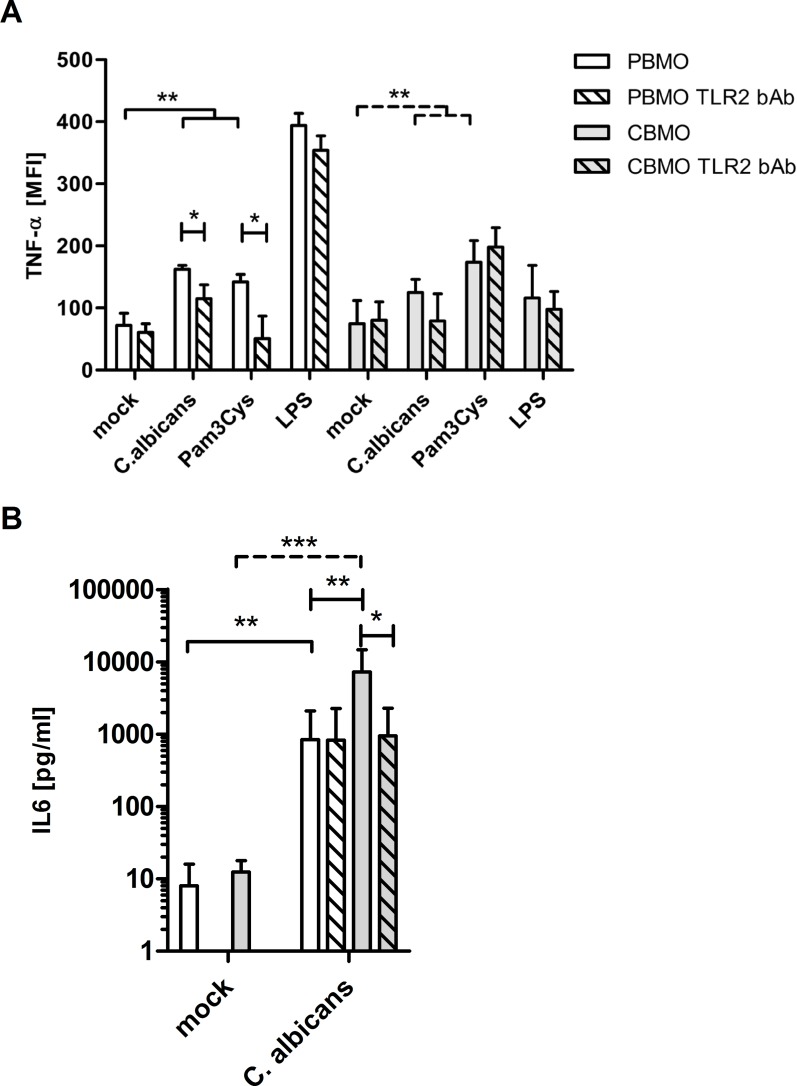
*C*. *albicans* induced TNF and IL-6 expression in neonatal and adult monocytes. CBMO and PBMO were infected with *C*. *albicans* at a MOI of 5 for 4h, or were treated with the indicated TLR agonists. The role of TLR2 for intracellular TNF-α expression was determined by administration of a TLR2 bAb (A, n = 5; 1-way ANOVA solid/dashed crotched lines, **p<0.01; 2-way-ANOVA blunt-ended lines and stars within bars, *p<0.05;**p<0.01; ***p<0.005). IL-6 secretion 4 h p.i. with or without TLR2 bAb administration as indicated (B, n = 3; student`s t-test solid/dashed crotched lines, **p<0.01; 2-way-ANOVA blunt-ended lines,*p<0.05; **p<0.01).

Phosphorylation of the P38 MAPK promotes downstream-signalling via JNK and NFκB, resulting in enhanced expression of cytokines such as TNF-α [14]. We, therefore, analyzed the intracellular TNF-α 4 h p.i. with *C*. *albicans* or following stimulation with TLR2 and TLR4 agonists (Fig 4A).

Basal intracellular TNF-α concentrations were comparable in PBMO and CBMO. Infection with *C*. *albicans* increased intracellular TNF-α levels in PBMO and CBMO. Pam3Cys also enhanced intracellular TNF-α concentrations. LPS led to higher intracellular TNF-α concentrations more dominant in PBMO than in CBMO (p < 0.05 vs. CBMO).

The requirement of TLR2 was confirmed by administration of a TLR2 bAb, which significantly reduced the *C*. *albicans*- as well as Pam3Cys-induced TNF-α production in PBMO. In CBMO, the addition of TLR2 bAb did not decrease the TNF-α production. The specificity of the TLR2 bAb was demonstrated by the absence of a detectable effect after administration of LPS (Fig 4A, 4^th^ columns). For *C*. *albicans* infected PBMO, the TLR2-dependency of TNF-α secretion could also be demonstrated by ELISA analysis (135.3.5 ± 23.4 pg/ml to 90.6 ± 20.7 pg/ml; p < 0.05 vs. TLR2 bAb treated).

Assessment of IL-6 secretion 4 h p.i. showed an almost 10 times stronger production of IL-6 in CBMO compared to PBMO (Fig 4B). Furthermore, the secreted IL-6 was diminished after application of a TLR2 bAb, whereas TLR2 signalling was not obligatory for IL-6 secretion in PBMO.

### *C*. *albicans* infection activates caspase-8 and caspase-3 via TLR2 to drive apoptosis in PBMO but not CBMO

**Fig 5:**
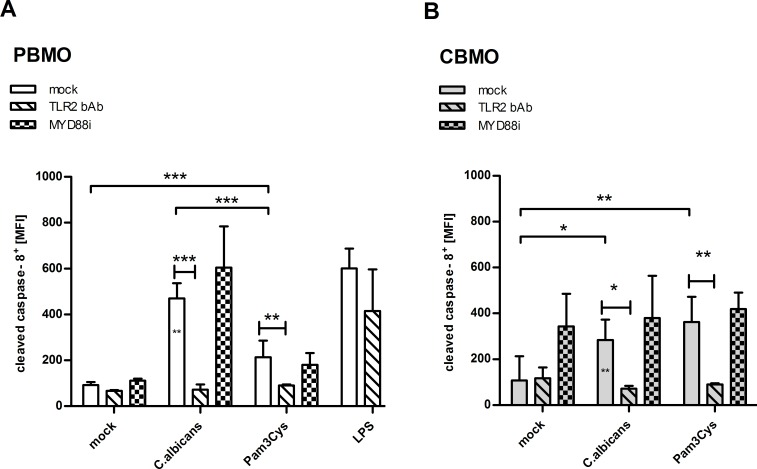
*C*. *albicans* infection-induced caspase-8. Mean fluorescence intensity (MFI) of the intracellular staining of cleaved caspase-8 in CD14^+^ PBMO (A) or CBMO (B) following infection with *C*. *albicans* at a MOI of 1:5. Pre-treatment with TLR2 bAb or MyD88i peptide (n = 5; student`s-t-test marked by solid (PBMO groups) or crotched (CBMO groups) lines, *p<0.05, **p<0.01, ***p<0.005; 1-way_ANOVA marked by blunt ended lines, *p<0.05, **p<0.01, ***p<0.005; 2-way-ANOVA marked by asterisks in chart bars, **p<0.01).

**Fig 6:**
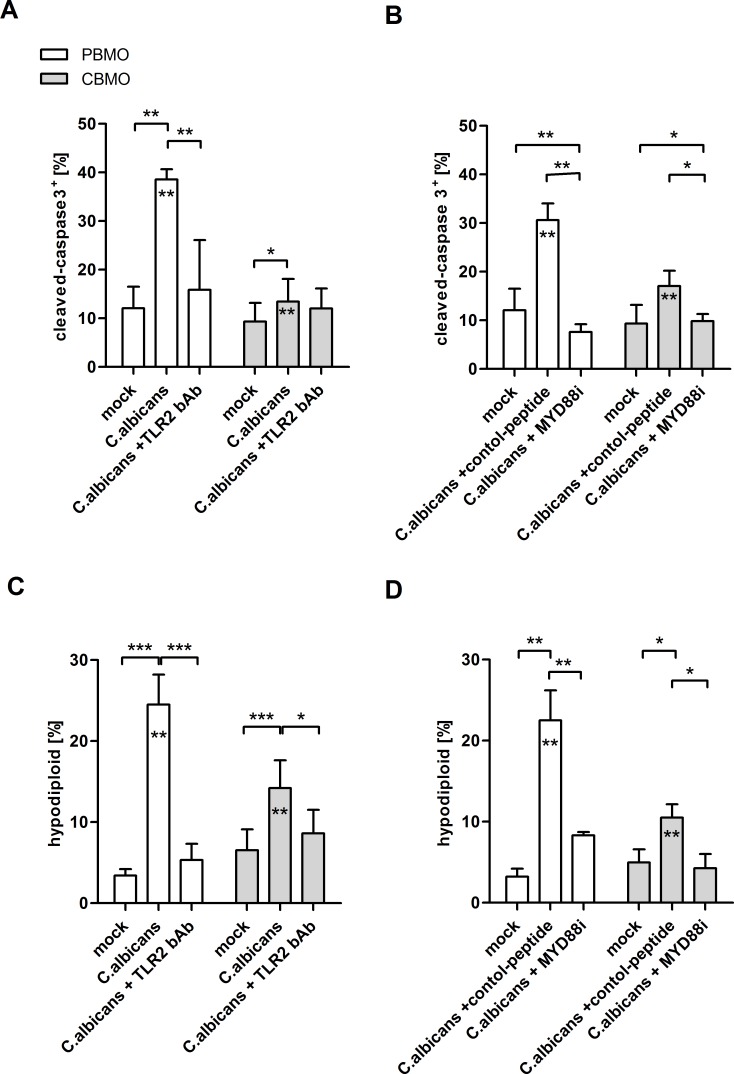
*C*. *albicans* infection initiates caspase-3 activation and monocyte apoptosis. Percentage of cleaved caspase-3 positive cells determined by flow cytometry (A, B; n = 5). Indicated groups were pre-treated with a TLR2 bAb (A) or MYD88i peptide (B) prior to infection with *C*. *albicans* at a MOI of 1:5. Percentage of hypodiploid (apoptotic) PBMO and CBMO (C and D) cells, following exposure to *C*. *albicans* at a MOI 1:5. Indicated groups were pre-treated with TLR2 bAb (C) or MYD88i peptide (D) prior to stimulation (n = 7, (A-D); 1-way-ANOVA marked by crotched lines, *p<0.05, **p<0.01, ***p<0.005; 2-way-ANOVA marked by asterisks in chart bars, **p<0.01).

Activation of caspase-8 by the TLR1-/TLR2-/MYD88-signalling may induce apoptosis [23]. We, therefore, tested whether caspase-8 and caspase-3 were cleaved to generate the active forms after *C*. *albicans* infection in PBMO and CBMO 2 h after infection (Figs 5 and 6). In PBMO cleaved caspase-8 products were found more prominently as compared to CBMO (Fig 5A and 5B; p < 0.05). The increase in caspase-8 cleavage was dependent on TLR2 and could be abrogated by pre-incubation with the TLR2 bAb. The TLR2/TLR1 agonist Pam3Cys initiated only a weak TLR2-dependent caspase-8 cleavage.

MYD88 was not engaged in the TLR2-induced caspase-8 activation, since MYD88 inhibition had no effect on caspase-8 cleavage (Fig 5). In PBMO but not CBMO, cleavage of effector caspase-3 was initiated by *C*. *albicans* infection (Fig 6A and 6B). Again, the involvement of TLR2 in caspase-3 activation was shown by administration of the TLR2 bAb (Fig 6A). Addition of MYD88 inhibitory peptide abrogated activation of caspase-3 (Fig 6B)

### *C*. *albicans* infection initiates apoptosis more dominantly in PBMO than in CBMO

Induction of apoptosis in *C*. *albicans* infected monocytes was assessed by measuring processing of caspase-3 (Fig 6A and 6B) determining the hypodiploid DNA content (Fig 6C and 6D) and detecting DNA strand breaks by TUNEL staining (Table 1). PICD was increased 5-fold following *C*. *albicans* infection in PBMO (Fig 6C), but only 2-fold in CBMO (Fig 6A; p < 0.05). Apoptosis in PBMO and CBMO was reduced to the levels of non-infected controls by administration of the TLR2 bAb, demonstrating the critical role of TLR2 (Fig 6C). Also, inhibition of MYD88 reduced monocyte apoptosis (Fig 6D), demonstrating the requirement of MYD88 signalling in TLR2-induced apoptosis.

**Table 1:** *C*. *albicans*-induced monocyte apoptosis.

	Mock	*C*. *albicans*
PBMO	1.74 ± 0.28 **	58.92 ± 20.45 **, #
CBMO	2.38 ± 0.15 *	14.5 ± 8.5 *, #

PBMO and CBMO were infected with *C*. *albicans* for 2h at the indicated MOI and analyzed following TUNEL staining. n = 3

*^,#^p<0,05

^#,^**p<0,005.

## Discussion

The present work describes marked differences in the activation of P38 MAPK phosphorylation, TNF-α production, caspase-8 and -3 processing, and phagocytosis induced cell death (PICD) in response to *C*. *albicans* infection between CBMO and PBMO despite similar phagocytosis capacity. The critical involvement of TLR2 in the recognition of *C*. *albicans* by PBMO suggests that reduced innate immune sensitivity might be responsible for the enhanced susceptibility of preterm neonates to fungal infection.

The phagocytosis of non-opsonized *C*. *albicans* by polymorphonuclear cells was previously demonstrated to be low in both, adult and neonatal neutrophils (10–12%), leading to the conclusion, that dysfunctional phagocytosis may not contribute to the increased susceptibility of preterm neonates to infection with *C*. *albicans* [24]. This is consistent with reports on similar phagocytic properties of PBMO and CBMO in respect to various bacterial species with pathogenic potential in the neonate (*E*. *coli*, Group B streptococci) [25].

During the first few weeks after birth, the TLR2 and TLR4 receptor densities on blood monocytes of human preterm newborns increases rapidly to reach levels that are similar to those of full-term newborns and adults [26, 27]. This is in line with our results on the expression of TLR2 and TLR4 by non-stimulated PBMO and CBMO (Fig 2A and 2C). Clinical investigations [28] and animal studies [29, 30] have demonstrated reduced TLR2 and TLR4 expression and diminished stimulus-induced cytokine secretion at birth both in preterm infants and term newborns. Our results demonstrating reduced TLR2 expression in CBMO are well in accordance with these studies. Though present work shows altered innate immune response against *C*. *albicans* in healthy full-term neonatal compared to adults. It seems that innate immunity is differentially regulated among neonatal subsets, depending on gestational age and birth weight, involving pattern recognition receptor- and TLR4-signalling [31, 32].

Upon recognition of the cognate ligand, TLRs activated the NFκB and MAPK signalling pathway leading to the expression of pro-inflammatory cytokines. In contrast to TLR2 which requires MYD88 for signal transduction, TLR4 can alternatively engage TRAM/TRIF [5, 32]. We observed that MYD88, a protein critically involved in TLR2 signalling, is abundantly expressed and stands by to drive downstream signalling in CBMO (Fig 3B). Other groups reported that MYD88 mRNA levels in CBMO are unaltered but proteins levels are slightly decreased [33, 34].

In accordance with previous studies [33] we observed a reduced MAPK P38 phosphorylation in CBMO after administration of a TLR2 ligand or *C*. *albicans*. In the published literature, the secretion of several pro-inflammatory cytokines such as IL-1ß, IL-6, IL-8 and TNF-α downstream of P38 MAPK activation was reported after stimulation of PBMO and CBMO. The results, however, have remained controversial. Some studies found the production of moderately less TNF-α, but similar or even higher IL-1 and IL-6 levels in CBMO as compared to adult monocytes [35], as it was shown here (Fig 4B). Other studies reported on a generally lower cytokine production [33].

Our results blend into these findings since infection with *C*. *albicans* resulted in an increased TNF-α production in PBMO (Fig 4). Stimulation of TLR2/TLR1 by Pam3Cys also induced significant TNF-α production in PBMO and CBMO (Fig 4) although TLR2 was found to be stronger up-regulated in PBMO (Fig 2B). Moreover, neutralizing TLR2 did not inhibit Pam3Cys initiated TNF-α production in CBMO. Additionally, MYD88 inhibition abrogated P38 phosphorylation completely after *C*.*albicans* infection but only partially after TLR2/TLR1 stimulation by Pam3Cys (Fig 3B and 3C), suggesting that P38 phosphorylation and TNF-α production in PBMO requires the engagement of co-receptors. Recent publications reported on a functional cross-talk between TLR2 and Dectin-1 [7, 36], which may account for the mismatching effects of TLR2 neutralizing antibodies and MYD88 inhibition. In CBMO, P38 phosphorylation can be blocked by a MYD88 inhibitor and a neutralizing antibody. Hence there is evidence for another P38-independent activation pathway that explains the intracellular TNF-α levels in CBMO after *C*.*albicans* infection and TLR2/TLR1 stimulation (Fig 4).

Although, the present study cannot thoroughly rule out a contribution of TLR4, which is down-regulated after *C*. *albicans* infection (Fig 2B), we suggest a minor role of TLR4 signalling. We could show that administration of LPS led to an ineffectual activation of P38 in comparison to the TLR2 agonists (Fig 2A). The TNF-α production, however, did not differ between TLR2/TLR1 stimulation and *C*. *albicans* infection (Fig 4), delimiting the role of TLR4. Several studies on foetal cells [26] and on cells obtained during the early postnatal period [27] indicate that the level of TLR4 expression does not correlate with TNF-α production. Albeit publications describe functional TLR2/TLR4 heterodimers which could initiate cytokine production, the designated TLR2/TLR4 heterodimer is formed after brain damage and exposure to haemoglobin [37].

TLR2 was one of the first TLR family members to be described as a death inducing receptor following overexpression in various cell types such as macrophages and neutrophils [14]. Pro-apoptotic signalling involves MYD88-dependent recruitment of FADD and caspase-8 leading to cleavage of caspase-3 and executing the apoptotic program [23, 38]. The pro-apoptotic and the cytokine-inducing signal transduction pathway downstream of TLR2 converge at the level of the P38 MAPK, which activates caspase-7 and -3. This may involve activation of the apoptosis-signal-regulating-kinase-1 (ASK1) leading to sustained P38 MAPK phosphorylation and activation of caspase-3 [14]. In previous studies, the involvement of TLR2 in apoptosis induction was established using blocking anti-TLR2 antibodies or comparison between TLR2 sufficient and deficient cells. More recently, the participation of TLR2 also in virus-triggered apoptosis was demonstrated [39].

We observed a pronounced TLR2-dependent activation of caspase-8 and caspase-3 in PBMO resulting in a *C*. *albicans*-mediated monocyte apoptosis (Figs 5 and 6). Caspase-8 activation could be reduced by TLR2 neutralizing antibodies but were resistant to MYD88 inhibition, suggesting an indirect contribution of TLR2. We suggest a trans-activation of caspase-8 which is caused by TLR2/Dectin-1 triggered TNF-α production (see above). Ligation of TNF-α to TNFR1, in turn, activates caspase-8, which is supported by the finding that TNF-α inhibitory antibodies reduce caspase-8 activation (unpublished results). The TLR2 ligand Pam3Cys induced caspase-8 equally in CBMO and PBMO, but exposure to *C*. *albicans* induced caspase-8 stronger in PBMO, pointing again to a contribution of a TLR-2 co-receptor.

The question that remains to be clarified is whether trans-activated caspase-8 contributes to caspase-3 activation and apoptosis. Here, we observed effector caspase-3 activation after *C*. *albicans* infection (Fig 6A), which could be blocked by TLR2 neutralizing antibodies and MYD88 inhibition, demonstrating a direct requirement of TLR2. Further studies should thus identify signal transducing factors linking P38 phosphorylation and activation of the intrinsic apoptotic machinery.

The reduced PICD of CBMO following *C*. *albicans* infection (Fig 5C) is consistent with our previous report on the response of cord blood monocytes to bacterial pathogens [15, 21]. Although a similar phagocytic index was detected in PBMO and CBMO following exposure to *E*. *coli* and GBS a reduced level of PICD was noted in CBMO. The results further point towards a crucial role of CD95L [15, 21] and TNF-α in these processes [16]. Together, this indicates age-dependent differences in the lifespan of macrophages following exposure to both bacterial and fungal pathogens although different receptor pathways appear to be involved. Since monocytes represent a major source of pro-inflammatory cytokines such as TNF-α or IL-6 during infection, this may explain the prolonged pro-inflammatory response and sustained inflammation in organs such as brain, lung and eye in neonates suffering from bacterial or fungal infection.

## Supporting Information

S1 Fig Assessment of apoptosis and gating strategies. Monocytes were infected with *C*. *albicans* with a MOI of 5. Apoptosis was assessed by detection of hypodiploid genomes of leukocytes (A, Nicoletti assay). Our gating strategy suspended both, yeast cells (red cells in the dot plot) and dublets of leucocytes (black cells) from analysis of DNA content by PI. Typical DNA content profiles of yeasts (left histogram), *C*. *albicans* infected leukocytes (right histogram) and non-infected leucocytes (right, lower histogram) reveals leukocytes with hypodiplod DNA (note the gate in the histograms to the right) after infection indicating induction of apoptosis. Alternatively, results of the Nicoletti assay were confirmed performing TUNEL assays (B). Infections with *C*. *albicans* increased the population of TUNEL-positive PBMC (compare upper left and right dot plots) compared to CBMO after infection (compare upper and lower dot plots to the right). DNAse treated CBMO (red overlaying dots in the lower left plot) served as positive controls. (C) Density plots and histogram plots of TLR2 stained monocytes before and after infection as indicated. Markers inserted in the histogram plots give a typical percentage of TLR2 expressing monocytes and numbers below represent MFI values. Dotted lines in the second left histogram plot represent the TLR2 expression of uninfected PBMO given to the left. (D) Dot plot analysis of PBMO before and after infection with *C*. *albicans* as indicated. Black dots represent PBMO stained with an iso-antibody to TLR2, grey dots represent PBMO stained with TLR2 antibody.
(TIF)

S2 Fig Assessment of mean TLR2 and TLR4 expression. Monocytes were infected with *C*. *albicans* (MOI 5) for 2h or were treated with indicated agonists. Left panel shows MFI assessment for TLR2 and the right panel MFI assessment for TLR4 (n = 5).
(TIF)

S3 Fig Assesment of P38 MAPK phosphorylation. PBMO and CBMO were treated for the indicated time intervals with 1 μg/ml Pam3Cys and subjected to intracellular staining of phosphorylated P38 MAPK as assessed by FACS analysis (n = 3; student`s-t-test. *p<0.05, **p<0.01, ***p<0.005; solid crotched bars, PBMO values, dotted crotched bars CBMO values).
(TIF)

## Abbreviations

*C. albicans*: Candida albicans
CBMO: cord blood mononuclear cells
DAPI: 4’,6-Diamidino-2-phenylindole-dihydrochloride
*E. coli*: *Escherichia coli*
FSL-1: diacylated lipopeptide derived from *Mycoplasma salivarium*
GFP: green fluorescent protein
GBS: Group B *Streptococcus*
IL-6: interleukin-6
LPS: Lipopolysaccharide
MOI: multiplicity of infection
MYD88i: inhibitory peptide, blocking MYD88 signalosom formation
P38 MAPK: P38 Mitogen-activated-protein Kinase
Pam3Cys: Pam3Cys4, triacylated (bacterial) lipoprotein
PBMO: peripheral blood monocytes from adults
PBS: phosphate buffered saline
PICD: phagocytosis induced cell death
PI: propidium iodide
p.i.: post infection
RT: room temperature
TLR2 bAb: Toll-like-receptor-2 blocking antibody
TNF-α: Tumor necrosis factor-alpha
