# Supplementary figures and images for: Reduced PICD in Monocytes Mounts Altered Neonate Immune Response to Candida albicans
Source: PLoS One. 2016 Nov 21;11(11):e0166648. doi: 10.1371/journal.pone.0166648 (PMC5117704; doi:10.1371/journal.pone.0166648)

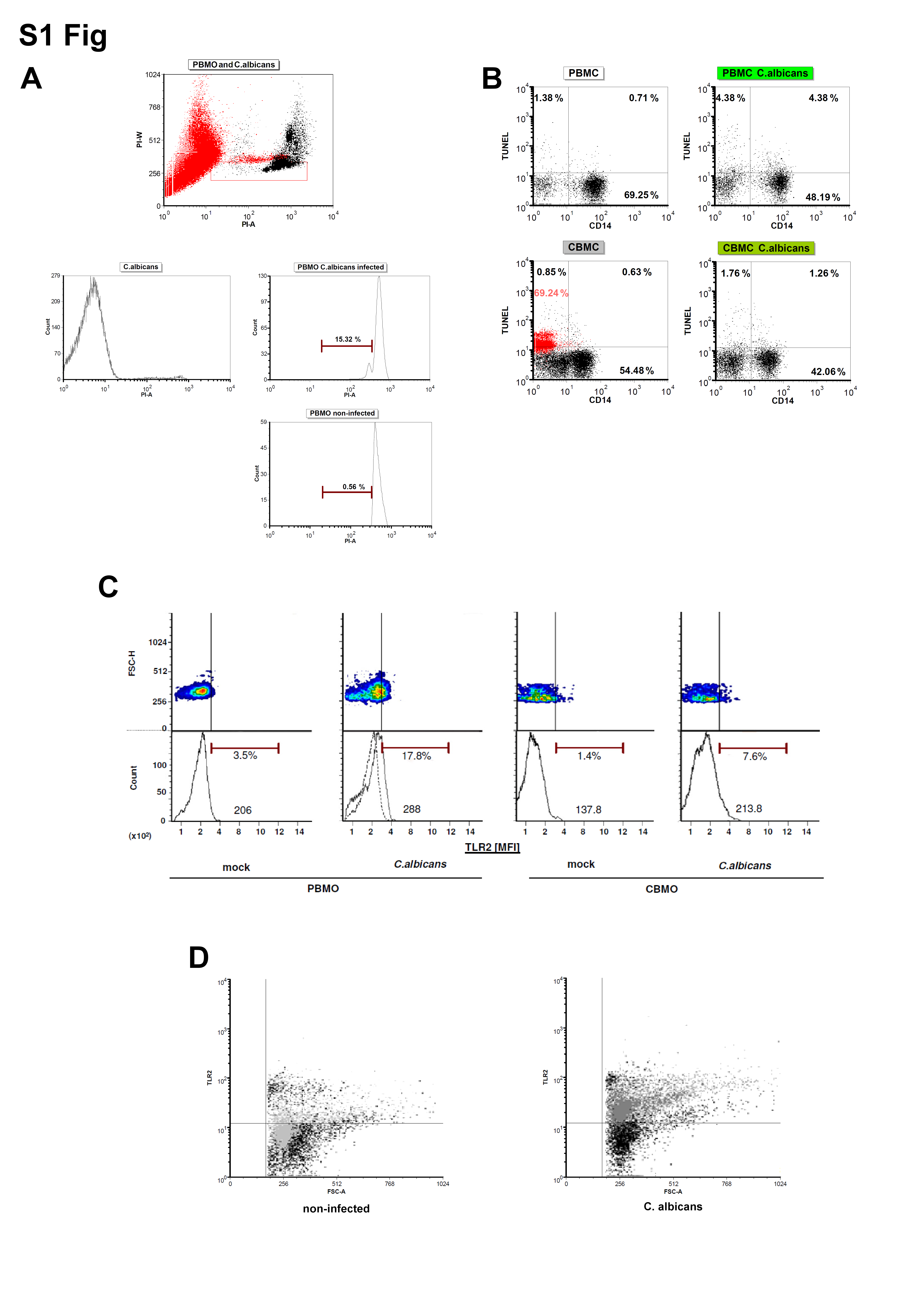

Supplement: S1 Fig — Monocytes were infected with C. albicans with a MOI of 5. Apoptosis was assessed by detection of hypodiploid genomes of leukocytes (A, Nicoletti assay). Our gating strategy suspended both, yeast cells (red cells in the dot plot) and dublets of leucocytes (black cells) from analysis of DNA content by PI. Typical DNA content profiles of yeasts (left histogram), C. albicans infected leukocytes (right histogram) and non-infected leucocytes (right, lower histogram) reveals leukocytes with hypodiplod DNA (note the gate in the histograms to the right) after infection indicating induction of apoptosis. Alternatively, results of the Nicoletti assay were confirmed performing TUNEL assays (B). Infections with C. albicans increased the population of TUNEL-positive PBMC (compare upper left and right dot plots) compared to CBMO after infection (compare upper and lower dot plots to the right). DNAse treated CBMO (red overlaying dots in the lower left plot) served as positive controls. (C) Density plots and histogram plots of TLR2 stained monocytes before and after infection as indicated. Markers inserted in the histogram plots give a typical percentage of TLR2 expressing monocytes and numbers below represent MFI values. Dotted lines in the second left histogram plot represent the TLR2 expression of uninfected PBMO given to the left. (D) Dot plot analysis of PBMO before and after infection with C. albicans as indicated. Black dots represent PBMO stained with an iso-antibody to TLR2, grey dots represent PBMO stained with TLR2 antibody. (TIF) [file pone.0166648.s001.tif]

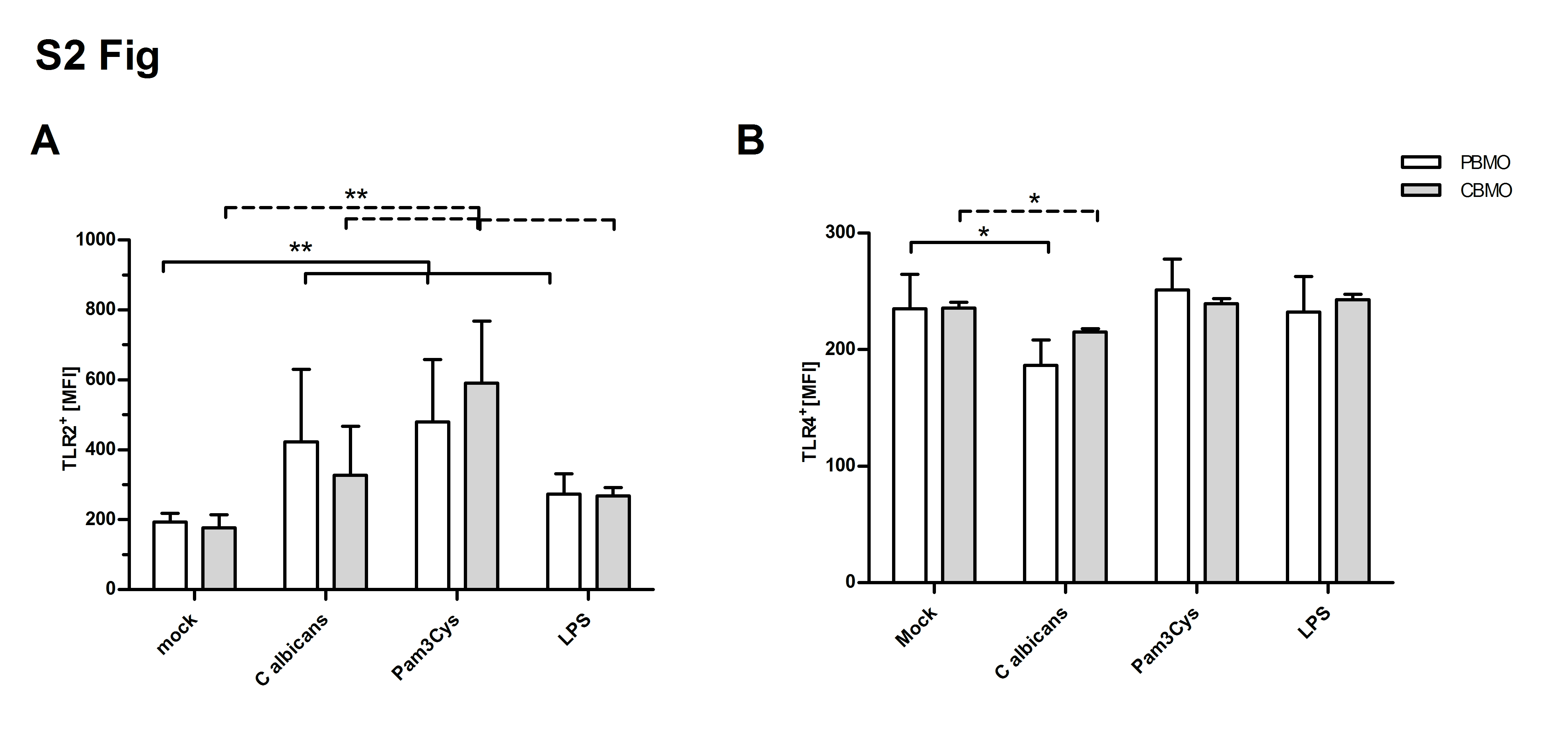

Supplement: S2 Fig — Monocytes were infected with C. albicans (MOI 5) for 2h or were treated with indicated agonists. Left panel shows MFI assessment for TLR2 and the right panel MFI assessment for TLR4 (n = 5). (TIF) [file pone.0166648.s002.tif]

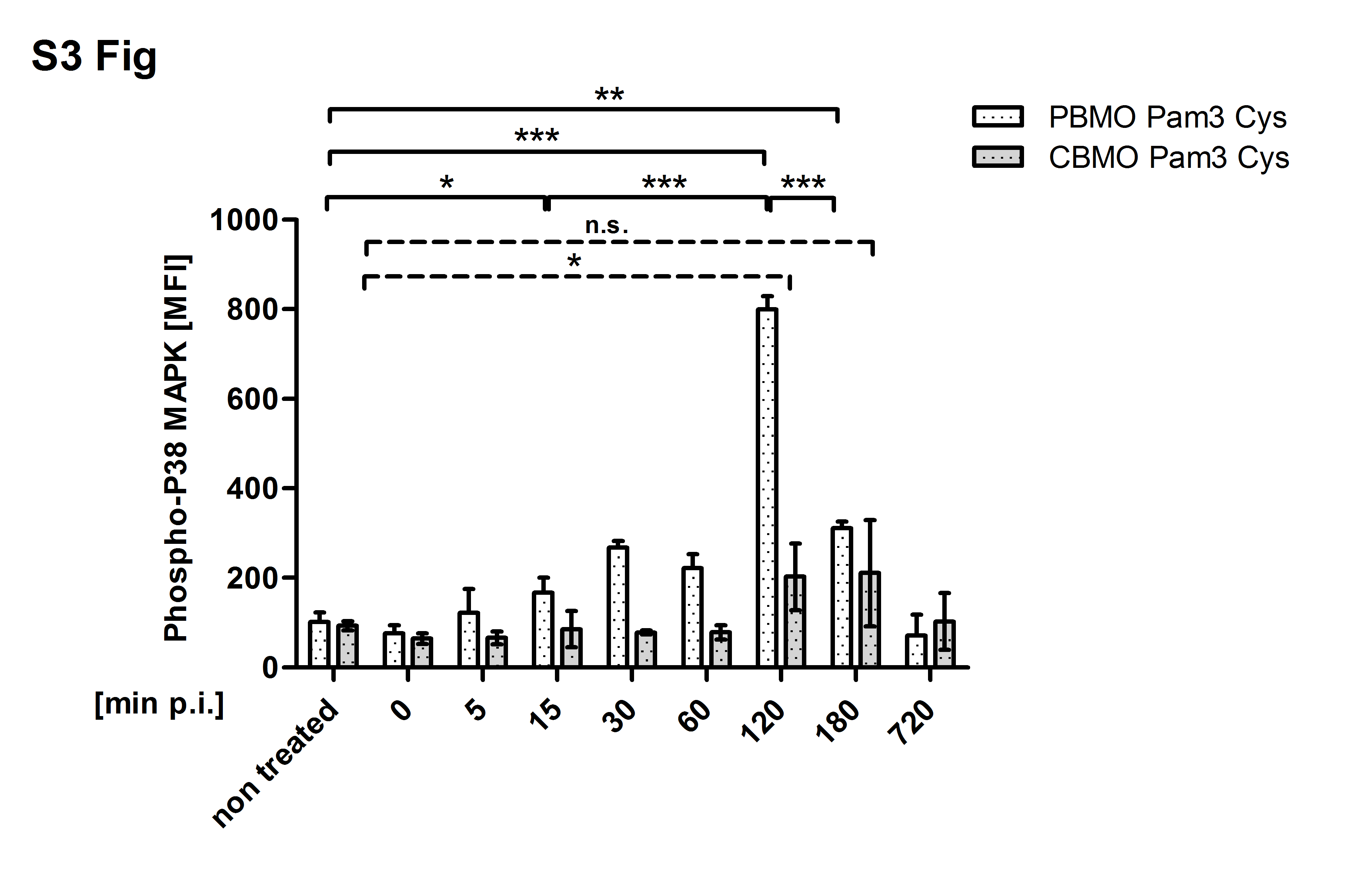

Supplement: S3 Fig — PBMO and CBMO were treated for the indicated time intervals with 1 μg/ml Pam3Cys and subjected to intracellular staining of phosphorylated P38 MAPK as assessed by FACS analysis (n = 3; student`s-t-test. *p<0.05, **p<0.01, ***p<0.005; solid crotched bars, PBMO values, dotted crotched bars CBMO values). (TIF) [file pone.0166648.s003.tif]
